# Outcome of patients with acute severe necrotizing pancreatitis in a dedicated hepato-biliary unit of Pakistan

**DOI:** 10.12669/pjms.37.3.3440

**Published:** 2021

**Authors:** Laima Alam, Rao Saad Ali Khan, Syed Kumail Hasan Kazmi, Rafi ud Din

**Affiliations:** 1 Laima Alam, FCPS Gastroenterology, Junior Consultant Gastroenterology, Bahria Town International Hospital, Rawalpindi, Pakistan; 2 Rao Saad Ali Khan, FCPS Medicine, FCPS Gastroenterology, Consultant Gastroenterology and Transplant Hepatologist, Pak Emirates Military Hospital, Rawalpindi, Pakistan; 3 Syed Kumail Hasan Kazmi, FCPS Medicine, Fellow Gastroenterology, Pak Emirates Military Hospital, Rawalpindi, Pakistan; 4 Rafi ud Din, FCPS Medicine, FCPS Gastroenterology, Consultant Gastroenterology, Combined Military Hospital, Quetta, Pakistan

**Keywords:** Acute pancreatitis, Acute necrotizing pancreatitis, Disease outcome, Infected pancreatic necrosis, Necrosectomy

## Abstract

**Objective:**

To analyze the management of severe necrotizing pancreatitis in a specialized center of a lower middle-income country, Pakistan using multiple outcome measures.

**Methods:**

All the patients in this prospective observational study with severe necrotizing pancreatitis being referred to Pak Emirates Military Hospital from January 2017 to December 2019 were followed over the course of their admission. Demographic data and disease outcomes were duly noted. Cox regression analysis was used to predict fatality outcome.

**Results:**

A total of 57 patients with 48 (84.6%) infected necrotizing pancreatitis were managed in our set up. The most common etiology reported was gall-stones (37%) with male preponderance (72%) and a mean age of 50±11.3 years. The most common complications were acute-kidney-injury (63%), splenic-vein-thrombosis (21%) and ascites (21%). Fourteen patients required mechanical-ventilation with a mean duration of 7±1.4 days on respiratory support. Eight (14%) patients required Endoscopic-Ultra-Sound guided drainage and six (10.5%) underwent surgical-necrosectomy depending upon the patients’ condition and collections characteristics. Mortality, as one of the main outcome measures, was reported to be 12.3% and was statistically related to mechanical-ventilation, organ failure and surgical-necrosectomy while 22 (38.6%) patients were discharged on pancreatic enzymes supplements and 7% required insulin.

**Conclusion:**

Survival outcomes with acute severe necrotizing pancreatitis are improving in a dedicated hepato-biliary unit internationally in lieu with a multidisciplinary team approach. Percutaneous and EUS guided drainage of pancreatic collections have turned out to be an important procedure to manage infected pancreatic necrosis that helps to avoid a morbid procedure in the form of necrosectomy.

## INTRODUCTION

Acute pancreatitis is a reversible inflammation of the parenchymal cells of pancreas and adjacent tissues, the management of which largely remains supportive. The reported incidence of acute pancreatitis ranges from 13 to 45 per 100,000 per annum^1^ with around 15% presenting as necrotizing pancreatitis, 42% of which become infected later on.^2^ Severity of the disease, multi-organ failure and the presence of infection in the necrotic tissue(s) and collection(s) are associated with increased morbidity and worse prognosis. The mortality of acute pancreatitis ranges from 4 to 25% with 50% of all deaths documented happening in the first 2 weeks of the acute insult and the rest due to multi-organ failure, infected necrosis, sepsis and in-hospital complications of acute pancreatitis.^3^

Pancreatic necrosis and fluid collections were historically treated surgically with poor outcome and significant mortality. Minimally invasive therapeutic options like percutaneous drainage, EUS guided drainage, video-assisted retroperitoneal debridement and endoscopic necrosectomy have emerged as reliable and efficacious interventions that are preferred over surgical necrosectomy in lieu of the changing predictors of outcome.^4^

A detailed literature review of the local data revealed multiple studies over the aetiology and severity indices of acute pancreatitis but only a few reported outcomes of surgically treated severe necrotizing pancreatitis and none detailed about minimally invasive strategies. To our best knowledge, this is the largest cohort of infected necrotizing pancreatitis being treated at a single dedicated center of Pakistan with multiple outcomes.

## METHODS

The data was for this prospective study was collected from January 2017 to December 2019 at Pak Emirates Military Hospital Rawalpindi, Pakistan and included all those patients treated in the hepato-biliary ICU for severe necrotizing pancreatitis. Ethical committee approval (Ref. No. A/28/179/EC/2020, dated July 28, 2020) was sought and written informed consent from the participants or next of kin was taken. Age, gender, aetiological factors and co-morbidities were noted for all patients. Patients with chronic pancreatitis, those presenting after three months of disease onset, those with mild or moderate severity pancreatitis and those who refused to be included in to the study were excluded.

### Definitions:

Pancreatitis was defined as the presence of any two of the three; acute abdominal pain, three times upper limit of normal (ULN) or more rise in serum amylase or lipase or radiological evidence of pancreatitis.^5^ All the plausible causes of pancreatitis including drugs/viral and autoimmune were sought. Severity of pancreatitis was defined according to the revised Atlanta classification whereas, pancreatic necrosis was diagnosed as ≥30% non-enhancing areas in pancreatic parenchyma on Contrast-Enhanced-CT.^3^ The size of the fluid or walled off collection was determined by measuring the largest cross-sectional diameter of the largest collection radiologically and obtaining the modified Computed Tomography Severity Index (CTSI).^3^ Infected necrosis was defined as culture positive aspirates from the collections^6^ or the presence of gas in the collection(s) on CT scan in the presence of systemic manifestation of sepsis.^5^ Recovery was defined as asymptomatic patients, tolerating oral feeds with normal inflammatory markers while off analgesia and antibiotics for three days.

### Drainage:

Difficult to manage pain, clinical deterioration, obstructive symptoms, and infected walled off collections and abscesses were the indications for drainage.^7^ Minimally invasive drainage (with drain size in the range 9-32Fr according to the collection size and consistency of the aspirates) was carried out by identifying the location of collection, catheter trajectory, effectiveness of drainage in relation to gravity and patient position, ease of wound care and avoiding restriction of movements in an ambulatory patient.^8^ Follow up ultrasound was used to document complete resolution of the collection(s) if the drain output was less than 20cc/24 hours, which was followed by drain removal. Endoscopic Ultrasound (EUS) guided transluminal drainage with placement of double pigtail stent (single or double 10F 2-3cm plastic stents, alternatively) was used as a step up procedure for minimal invasive drainage, followed by invasive surgical necrosectomy for those showing progressive organ dysfunction and deterioration on non-invasive regimen. The step-up regimen for non-invasive drainage was introduced due to resource limitation.

### Outcome:

The main outcome measures included recovery, in hospital complications (including infected/sterile necrosis, splenic vein thrombosis, pneumonia, ascites, single or multi-organ-failure, bleeding and gastric outlet obstruction), interventions; both minimally invasive and invasive, length of hospital stay, nutritional support, number of drains inserted, interval between drain placement and removal, use of intravenous antibiotics and mortality.

### Statistical analysis:

Data was expressed as mean ± SD, frequencies and percentages where appropriate. Chi square statistics were used for comparing qualitative data and cox regression analysis was used to predict fatal outcome in the study group. A p value of <0.05 was considered statistically significant. All data was analyzed using SPSS V. 19.

## RESULTS

A total of 57 patients with severe necrotizing pancreatitis were managed in this dedicated hepato-biliary set up. Male patients showed preponderance (72%) and the mean age for the participants was 50 ± 11.3 years with majority of the patients in 51-60 years range. The most common etiological factor for severe necrotizing pancreatitis was gall stones (37%), followed by idiopathic causes (17.5%) and smoking (16%). Majority of the patients’ pancreatitis severity landed in to CTSI score 10 (54.4%), with the most common mode of nutrition being NG feed (35%) (Table-I).

**Table-I:** Characteristics of severe necrotizing pancreatitis in the study population (n=57).

Variables	Frequency (n) or mean	Percentage (%) or ± SD
***Gender***		
Males	40	70
Females	17	30
***Age (years)***		
Mean age	50	11.3
21-30	3	5.3
31-40	8	14
41-50	15	24.3
51-60	22	38.6
61-70	9	15.8
***BMI (kg/m2)***		
15.1 – 20	2	3.5
20.1 – 25	17	30
25.1 – 30	37	65
30.1 – 35	1	2
***Aetiology***		
Gall stones	21	37
Idiopathic	10	17.5
Cigarette smoking alone	9	16
Post-ERCP	6	10.5
Cigarette smoking and gall stones	4	7
Hypertriglyceridemia and gall stones	4	7
Hypertriglyceridemia alone	2	3.5
Alcohol	1	2
***Comorbidities***		
None	26	45.6
Diabetes mellitus	10	17.5
Hypertension	10	17.5
Ischemic heart disease	1	2
Diabetes mellitus and hypertension	3	5.3
Diabetes mellitus and ischemic heart disease	2	3.5
Hypertension and ischemic heart disease	1	2
Diabetes, hypertension and ischemic heart disease	3	5.3
***CTSI scores***		
8	12	21
9	14	24.6
10	31	54.4
***Mode of nutrition***		
Oral feed only	3	5.3
NG feed only	20	35
Partial TPN with NG feed	16	28
NJ feed only	14	24.5
Partial TPN with NJ feed	3	5.3
TPN only	1	2

The median duration of stay at the hospital was 39 (IQR 15) days, with the most common complications being infection in pancreatic necrosis or peri-pancreatic collection(s) (84.2%), acute kidney injury (63%), splenic vein thrombosis (21%) and ascites (21%). Fourteen patients required mechanical ventilation with a mean duration of 7 ± 1.4 days (Table-II). Eight patients required EUS guided drainage as a step up procedure and six underwent surgical necrosectomy. None of the percutaneous drains were reported to be blocked due to meticulous irrigation with saline every eight hourly. Mortality, as one of the main outcome measures, was reported to be 12.3%, while 22 (38.6%) patients were discharged on pancreatic enzymes supplements and 7% required insulin.

**Table II:** Outcome measures for severe necrotizing pancreatitis in the study population (n=57).

Outcome variables	Frequency or mean	Percentage or ± SD
***Stay in hospital (days)***		
Median duration of stay in hospital	39	15 (Interquartile range)
***Mechanical ventilation***		
No of patients	14	24.5
Mean duration on vent (days)	7	1.4
-1 to 5 days on vent	8	14
-6 to 10 days on vent	6	10.5
Pancreatic insufficiency		
Patients on exocrine support upon discharge	22	38.6
Patients on endocrine support (insulin) upon discharge	4	7
***Number of drain(s)***		
1	3	5.3
2	17	30
3	15	26.3
4	15	26.3
5	5	9
6	0	0
7	1	2
8	1	2
***Interval between drain placement and removal (days)***		
6-10	1	2
11-15	12	21
16-20	12	21
21-25	11	19.3
26-30	9	16
31-35	7	12.3
36-40	2	3.5
>40	3	5.3
***EUS guided drainage***^£^		
No. of patients	8	14
Surgical necrosectomy*		
Total	6	10.5
Before 30 days	5	8.7
30 to 60 days	1	1.8
***Complications***		
Infected necrosis / peri-pancreatic collections	48	84.2
Pseudocyst	9	16
Sterile pancreatic necrosis	7	12.3
Splenic vein thrombosis	12	21
Pneumonia	9	16
Ascites	12	21
Acute respiratory failure	5	8.8
Circulatory failure	2	3.5
Acute kidney injury	36	63
Multi-organ failure	7	12.3
Bleeding	2	3.5
Gastric outlet obstruction	2	3.5
***Mortality***		
No of deaths	7	12.3
1-Post ERCP pancreatitis	2	3.5
2-Idiopathic pancreatitis	2	3.5
3-Gall stone pancreatitis	3	5.3

£ EUS guided drainage was statistically related to lower requirement of percutaneous drains (p=0.03).

* Surgical necrosectomy was statistically related to mortality (p<0.001), need for mechanical ventilation (p<0.001), less number of percutaneous drains (p<0.001) and shorter duration of drains in-situ (p=0.006)

All the patients were given intravenous antibiotics and the aspirates from 48 patients grew *E.coli*, Klebsiella, Pseudomonas and mixed organisms in descending order of frequency (Table-III) and majority of the antibiotics showed reasonable sensitivity towards targeted micro-organisms.

**Table III:** Organisms grown with their sensitivities from pancreatic and peri-pancreatic collections (n=48).

Microbes	Culture positive aspirates n(%)	Sensitivity n(%)					

		Piperacillin/ Tazobactam	Meropenem	Colistin	Ciprofloxacin	Amikacin	Tigecycline
*E coli*	29 (60.4)	23 (79.3)	28 (96.6)	28 (96.6)	4 (14)	1 (3.4)	8 (27.6)
*Klebsiella*	20 (41.7)	13 (65)	18 (90)	18 (90)	4 (20)	0	11 (55)
*Psuedomonas*	15 (31.3)	9 (60)	15 (100)	15 (100)	1 (6.7)	1 (6.7)	9 (60)

In the univariate regression analysis, severity of pancreatitis, need for mechanical ventilation, surgical necrosectomy, in-hospital complications and the presence of co-morbidities were statistically significant (p<0.05) for predicting mortality. Whereas in the final model using binary logistic regression analysis, only surgical necrosectomy acted as independent predictor of mortality. Need for mechanical ventilation, surgical necrosectomy, circulatory collapse and multi-organ failure were the determinants of mortality through Cox Regression Analysis (Table-IV).

**Table-IV:** Results of Cox regression analysis for fatal outcome.

Variables	Hazard ratio (CI 95%)	P value
Age	1.06 (0.98 – 1.14)	0.15
Gender	2.82 (0.34 – 23.48)	0.34
BMI	2.17 (0.48 – 9.81)	0.31
Etiology	1.35 (0.93 – 1.97)	0.11
Comorbidities	59.8 (0.14 – 264.6)	0.19
CTSI	17.67 (0.427 – 112.7)	0.18
Mechanical ventilation	16.86 (2.21 – 128.56)	0.006
EUS guided drainage	0.04 (0.00 – 389.3)	0.49
Surgical necrosectomy	40.96 (7.75 – 216.47)	<0.001
Infected pancreatic necrosis	23.46 (0.00 – 87.39)	0.58
Any organ failure	41.18 (0.67 - 9.55)	0.26
AKI	43.4 (0.07 – 2.46)	0.24
Respiratory failure	4.91 (0.95 – 25.36)	0.06
Circulatory failure	30.57 (6.0 – 155.52)	<0.001
Multi-organ failure	28.63 (2.93 – 279.3)	0.004

## DISCUSSION

Acute pancreatitis has been following an increasing trend during the last 40 years with an incidence of 20 to 80 cases per 100,000 population per annum in some countries.^9^ Infected necrotizing pancreatitis is a potentially life threatening disease with step up management protocol being advocated by many.^10^ The morbidity and mortality outcomes in severe necrotizing pancreatitis are governed by the presence of infected necrosis, sepsis and multi-organ failure.^11^ With changing management strategies and early recognition of infection, clinical outcome of necrotizing pancreatitis is changing.^12^

### Demographics:

Our set-up managed 57 patients of severe necrotizing pancreatitis, 84.2% of whom had infected pancreatic necrosis (IPN) or peri-pancreatic collection(s). The aetiology, male preponderance and middle age in our study were advocated by many similar studies.^3^,^13^ A study by Lee JK et al explained that genetic testing for the previously labelled idiopathic pancreatitis after an extensive panel of investigations might explain the aetiology in many of the patients, although many of these genetic disorders have limited therapies, adding to the patients’ anguish.^14^,^15^

### Severity and outcomes:

Majority of the patients managed at our set up, being a tertiary referral hospital, had a high CTSI score that correlated to higher number of drains and longer duration of drains insertion and related in-hospital complications. Similarly, the requirement for mechanical ventilation and step up approach was seen with higher CTSI scores and in patients with co-morbidities.

A prolonged hospital stay is associated with increased in-hospital complications including nosocomial infections, thrombo-embolic phenomenon, malnutrition and excessive economic burden. The median stay for our cohort was 39 (15 IQR) days which was lower than a similar study that reported a range of three to 120 days on average^3^. Our study reported a relatively shorter interval between the insertion and removal of drain(s) as compared to similar outcome studies (29.83±25.58 days)^2^ probably because of the multiple drain’s insertion regimen for efficient drainage and a prompt step up approach whenever required.

Pancreatic insufficiency after severe parenchymal insult is not uncommon and has been reported to be as high as 35% to 62% for exocrine and 23% for endocrine insufficiency.^10^ The trend showed an upslope for severe necrotizing pancreatitis and a slow recovery in two-thirds of the patients in a meta-analysis by Huang W et al.^16^ No data was available from the native country regarding this important sequelae.

In order to minimize translocation of bacteria and the incidence of infection in pancreatic necrosis, enteral nutrition has always been considered paramount to help with sustaining intestinal mucosal barrier. Also, pancreatic rest that is achieved with TPN is sometimes required for severe necrotizing disease.^14^ Thirty-three percent of our patients required combined enteral and partial TPN for their nutritional requirements whereas only 5.3% were able to tolerate only oral intake during their ICU stay. An indigenous study reported 44.7% of the patients receiving enteral feed and 49% kept nil per os (NPO) with only 8.2% receiving TPN and none on oral feed alone.^17^

### Mortality:

The mortality (12.3%) for our set up was lower as compared to many studies, with one review article estimating the total mortality of acute severe pancreatitis as 13.6% to 41.9%.^13^ Mortality was seen to be statistically associated with surgical necrosectomy mainly because of the morbidity and strong inflammatory response associated with surgical manipulation, leading to further deterioration of the failing organ systems. A local study reported a mortality of 26% post-surgical necrosectomy that included only 6 (12%) patients with infected necrosis and multi-organ dysfunction.^18^ A study by Jain S et al. demonstrated a mortality of 43% in a large cohort of infected necrotizing pancreatitis, all of which were treated by minimally invasive drainage techniques and antibiotics.^3^

In the final model using binary logistic regression analysis, only surgical necrosectomy acted as independent predictor of mortality. The reason might be the fact that all the patients admitted had severe necrotizing pancreatitis with CTSI scores ≥8 (eliminating the temporal effect of severity on mortality) and the presence of relatively younger population in the study group.

### Complications:

The most common single organ failure in our study was acute renal failure (63%), that was reported to be 17% by van Brunschot S et al, with multi-organ dysfunction (17.5%) higher than our study.^19^ Only two patients in our cohort bled secondary to fundal varices on the background of splenic vein thrombosis and required sclerotherapy. Hyun J et al. reported bleeding as a complication to be 8%, gastrointestinal obstruction as 5.3% and biliary strictures as 3.5% in a study with 211 patients.^2^

### Antimicrobials and their sensitivities:

The two most important determinants of mortality outcome in acute necrotizing pancreatitis are infected collections and organ dysfunction.^3^ Mono-bacterial infections were reported more commonly than poly-bacterial by many researchers and a trend towards multi-drug resistant organisms and fungal infection is seen.^20^ All the patients in our center were given prophylactic antibiotics that were modified according to the culture and sensitivities. None of the infected pancreatic necrosis or peri-pancreatic collection yielded multi-resistant or gram-positive organism and the antibiogram showed reasonable sensitivities towards commonly used antibiotics in the ICU set up. A study by Jain S and colleagues demonstrated 127 multi-drug resistant cases in a sample of 149 pancreatic and peri-pancreatic isolates with *E.coli* being the most abundant organism.^3^ A prompt diagnosis and early treatment initiation with anti-microbes able to attain effective therapeutic levels in pancreatic tissue result in improved outcome in these patients.

### Limitations of the study:

An extensive literature review showed that none of the local studies highlighted the antibiograms, extensive follow up and multiple outcome measures as in our study. Quality of life of the patients, the economic burden secondary to work days lost and the single center data with no local database to compare outcomes with are some of the limitations. Nevertheless, this study features many important aspects of hospital management and outcomes that weren’t studied in detail previously, indigenously.

## CONCLUSION

Survival rate of patients with acute severe necrotizing pancreatitis is good in a dedicated hepato-biliary unit where there is a multidisciplinary team approach for management, including gastroenterologists, interventional radiologists and hepato-biliary surgeons. Percutaneous drainage of pancreatic collections has turned out to be an important procedure in resource deficient countries to manage infected pancreatic necrosis that helps to avoid a morbid procedure in the form of necrosectomy. Circulatory failure and multi-organ dysfunction are the two determinants of worse prognosis and need prompt diagnosis and aggressive management.

### Author`s Contribution:

**LA** contributed to the design of the manuscript, drafting, statistical analysis and literature review. **RSAK** contributed to the idea, patient care and data collection.

**SKHK** contributed to data collection.

**RD** contributed to critical review. All authors approved the final version to be published and agree to be accountable for all aspects of the work.
